# Implementation of a clinical interview guide to Multidimensional needs assessment in Palliative care (MAP): A multicenter mixed-methods feasibility study

**DOI:** 10.1371/journal.pone.0329354

**Published:** 2025-07-31

**Authors:** Iris Crespo, Blanca Goni-Fuste, Cristina Monforte-Royo, Aina Garcia-Salanova, Andrea Rodríguez-Prat, Alberto Alonso-Babarro, Margarita Alvaro, Pierluigi Bavestrello, Alazne Belar, David Bottaro, Diego Candelmi, Elisabet Casas, Emma Costas-Muñoz, Claudia Cruz Sequeiros, Natalia de Iriarte, Ana De Santiago, Jennifer Garrillo, Jesús González-Barboteo, Maria Jimeno Ariztia, Maria Nabal Vicuña, Lina Nitola-Mendoza, Pablo Noguera-Sánchez, Javier Rocafort, Dulce Rodríguez, Carme Sala, Judith Serna, Dolors Torremorell, Albert Balaguer, Joaquim Julià-Torras

**Affiliations:** 1 Department of Psychology, School of Medicine and Health Sciences, Universitat Internacional de Catalunya, Barcelona, Spain; 2 Department of Nursing, School of Medicine and Health Sciences, Universitat Internacional de Catalunya, Barcelona, Spain; 3 Department of Humanities, School of Humanities, Universitat Internacional de Catalunya, Barcelona, Spain; 4 Hospital Universitario La Paz, Universitat Autónoma de Madrid, Madrid, Spain; 5 Palliative Care Department, Catalan Institute of Oncology, Badalona, Spain; 6 Palliative Care Department, Knowledge and Research Group on Palliative Care (GRICOPAL), Catalan Institute of Oncology, L’Hospitalet, Spain; 7 Instituto Cultura y Sociedad, Universidad de Navarra, IdiSNA, Pamplona, Spain; 8 Palliative and Supportive Care Team, Institut Oncològic Catalunya Sud (IOCS), Hospital Universitario Salut Sant Joan, Reus, Spain,; 9 Facultad de Medicina, Universitat Rovira i Virgili, URV Reus, Spain,; 10 Palliative Care Department, Clínica Universidad de Navarra, Pamplona, Spain,; 11 Palliative Care Service, Consorci Sanitari de Terrassa, Hospital Universitario Terrassa, Spain,; 12 Palliative Home Care Team (PADES) Mutuam, Spain,; 13 Unitat de Suport i Cures Pal·liatives Cuides, Universitat Internacional de Catalunya, UIC Barcelona, Sant Cugat del Vallès, Barcelona, Spain; 14 Palliative Home Care Team, Hospital Centro de Cuidados Laguna, Madrid, Spain; 15 Hospital Universitari Vall d’Hebron, Barcelona, Spain; 16 Palliative and Supportive Care Team, Hospital Universitario Arnau de Vilanova, Lleida, Spain,; 17 Facultad de Medicina, Universidad de Lleida. IRB LLEIDA, Spain,; 18 Palliative Care Unit, Hospital San Juan de Dios, Pamplona, Spain,; 19 School of Medicine, Universidad Francisco de Vitoria, Madrid, Spain,; 20 School of Medicine and Health Sciences, Universitat Internacional de Catalunya, Barcelona, Spain; Queensland University of Technology - QUT: Queensland University of Technology, AUSTRALIA

## Abstract

**Background:**

A recent systematic review highlighted the lack of consensus on the needs that should be assessed in palliative care to develop the initial therapeutic plan. An agreed clinical interview guide for Multidimensional needs Assessment in Palliative Care (MAP) has recently been proposed.

**Objective:**

To evaluate the feasibility of implementing the MAP guide in clinical practice.

**Methods:**

A multicenter explanatory sequential mixed-methods feasibility study was conducted, assessing five indicators: a) acceptability to patients and family members (assessed by phone); b) participation (proportion of eligible patients assessed); c) applicability (time to administer); d) clinical utility as perceived by physicians; and e) implementation in practice. Twenty-four palliative care physicians across 10 services (outpatient, in-patient, domiciliary care) administered the MAP guide in 239 initial assessments of patients with advanced cancer. A focus group was conducted with 17 of the physicians to gather insights.

**Results:**

Indicators of acceptability, participation, applicability, and perceived clinical utility were fulfilled in over 90% of interviews. Implementation fell just short of the criterion (78% of needs assessed vs. 80% threshold). Patients and families provided highly positive feedback on the appropriateness of the MAP guide. Physicians found it flexible and easy to integrate into clinical practice, helping them structure the initial assessment and offer a much more comprehensive assessment of patients’ needs.

**Conclusions:**

The study supports the feasibility of using the MAP guide to explore palliative care needs. The MAP guide can help ensure that professionals do not overlook unmet needs, which could increase suffering and undermine quality of life.

## Introduction

The initial assessment of needs in palliative care is important for drawing up an adequate care plan that can relieve or prevent suffering and improve quality of life [1]. Although various attempts have been made to systematize needs assessments in palliative care [2,3], the developed tools are not widely implemented in practice and fail to cover all needs. Indeed, many structured questionnaires, often self-administered [4], focus on physical symptoms [2,5]. This approach not only places an additional burden on the patient, but also, when combined with numerical rating scales, restricts opportunities for patients and their families to fully express their needs [6,7]. Moreover, patients are not always able to articulate their priorities without empathetic engagement from healthcare professionals [8]. This lack of meaningful interaction can hinder the development of a strong therapeutic relationship between the patient and the palliative care team. Unsurprisingly, therefore, there is evidence that the palliative care needs of patients and families often go unmet [9–11], resulting in emotional distress, poorer quality of life, increased health care costs, and potentially lower survival rates [12–16].

Palliative care professionals recognize the need for the systematic initial assessment of needs within open dialogues with patients and families [17,18]. If the palliative care encounter is to be a meaningful and satisfactory experience for the latter, it must facilitate the exploration and recognition of all their needs [19]. In geriatric care, comprehensive bio-psycho-social assessments are common and yield significant clinical benefits [20–24]. Palliative and geriatric care share key characteristics, including patient frailty, the potential for rapid decline, and complex, multidimensional needs. Therefore, conducting a systematic and comprehensive assessment of needs in palliative care is likely to provide similar benefits to those achieved in the geriatric setting.

With the aim of improving the initial assessment of palliative care needs our group recently developed a structured clinical interview guide known as MAP: Multidimensional needs Assessment in Palliative care [25]. The MAP guide was conceptualized based on specific multidimensional care models [26,27] and the development process followed Medical Research Council guidance for the development of complex clinical interventions [28], and it involved four steps which have been previously published: 1) systematic review of needs assessment in palliative care [5]; 2) exploratory qualitative study with patients, family carers, and palliative care professionals [19]; 3) nominal group with palliative care experts [25]; and 4) modified Delphi process involving palliative care physicians [25]. The complete study protocol has been published elsewhere [29]. Once the MAP guide was developed, the present study aimed to evaluate the feasibility of implementing the MAP guide in real clinical practice following the RE-AIM (Reach, Effectiveness, Adoption, Implementation, Maintenance) framework [30].

## Methods

This was a multicenter explanatory sequential mixed-methods feasibility study [31] comprising two steps:

Step 1 (quantitative component of the feasibility study): The MAP guide was trialed in 10 palliative care services (outpatient, in-patient, domiciliary) across Spain to determine its feasibility in real clinical practice. Services were selected using convenience sampling by contacting professionals known to the research team representing both large and small palliative care units from public, private and subsidized centers, to ensure maximum variability.

Step 2 (qualitative component of the feasibility study): A focus group was conducted with a sub-sample of the palliative care physicians who had applied the MAP guide in order to gain insight into their experience.

### Participants

Eligibility criteria for patients included: (a) age 18 or over, (b) advanced cancer (distant metastasis, life-limiting cancer and/or prognosis of 6–24 months), as defined by the American Society of Clinical Oncology [32], and (c) attending palliative care for the first time. Exclusion criteria were: (a) severe communication difficulties that prevented assessment of needs and/or (b) cognitive impairment (≥5 errors on the Pfeiffer questionnaire). Eligible family members were those present during the patient’s initial palliative care encounter and the MAP guide application. Physicians were eligible if they worked in palliative care (either outpatient, in-patient or domiciliary service) and had applied MAP.

### Procedure and intervention

We began by explaining the purpose of the study to physicians in each palliative care service and inviting them to participate. Those who accepted received brief training on using MAP, a clinical interview guide for systematically assessing needs to inform the initial therapeutic plan. The training included a video tutorial on how to use MAP, followed by a one-hour online session featuring role-playing and a Q&A segment. As seen in Table 1, the MAP guide covers 47 needs across six domains: Clinical history and medical conditions (8 needs), Physical symptoms (17 needs), Functional and cognitive status (4 needs), Psycho-emotional symptoms (5 needs), Social issues (8 needs), and Spiritual and existential concerns (5 needs) [25].

**Table 1:** Domains and needs assessed in the MAP guide.

Domains and needs assessed in the MAP guide
**D1: Clinical history and medical conditions**
• Patient’s understanding of reason for palliative care referral • Diagnosis → therapeutic interventions → current status of illness (timeline) • Expectations and impact of the current illness • Relevant medical history (including psychiatric/psychological disorders) • Past or current substance abuse • Previous surgery • Current pharmacological treatment • Current complementary/alternative therapies
**D2: Physical symptoms**
• Pain • General malaise • Asthenia or fatigue • Anorexia • Dry mouth • Nausea or vomiting • Dyspnea • Cough • Constipation • Urinary symptoms • Bleeding • Daytime sleepiness • Insomnia • Changes to sensitivity • Pruritus • Weakness/Paresis • Myoclonus
**D3: Functional and cognitive status**
• Degree of functional dependence • Cognitive status • Need for information about symptoms and their causes, treatment, and prognosis • Hallucinations (visual, tactile and/or auditory)
D4: Psycho-emotional symptoms
• Mood and affect • Anxiety • Depression • Current concerns/worries • Loneliness or social isolation
D5: Social issues
• Main caregiver • Perceived support (by caregiver and by patient) • Organization of care at home • Family tree (relationships) • Family conflicts • Level of open communication between patient and family • Architectural barriers in the patient’s home • Need for social care
D6: Spiritual and existential concerns
• Aspects that help the patient cope • Religious beliefs and/or practices • Things that give meaning to the patient’s life • Patient’s values in life • Aspects of life that are important to patient in current situation

The recruitment period started on 3 October 2022 and ended on 28 September 2023. Participating physicians used the MAP guide during the initial palliative care assessment (in the first or first and second appointments) of consecutive patients. Patients and their accompanying family member(s) were informed about the nature of the study at the start of the interview.

Following application of the MAP guide, physicians logged in the patient’s clinical records all the needs that had been explored. Researchers reviewed these records to document the explored needs. In addition, and subsequent to each needs assessment clinical interview, a member of the research team telephoned the patient (or family contact) to ask whether they considered the exploration of needs to have been appropriate.

All participants (physicians, patients and family members) signed informed consent prior to data collection.

### Feasibility indicators

Based on published recommendations [33] we established five indicators to evaluate the feasibility of using the MAP guide in clinical practice: Acceptability, Participation, Applicability, Utility, and Implementation. In the context of advanced cancer, we determined that a minimum of 75% of participants is required to ensure the feasibility of MAP [34]. Table 2 lists each of these indicators, along with their definition, the measure used to assess them, and the criterion for fulfillment.

**Table 2:** The five indicators (definition, measure, and fulfillment criterion) used to evaluate the feasibility of applying the MAP guide in clinical practice.

Indicator	Definition	Measure	Criterion
**Acceptability**	Opinion of patient and accompanying relative(s) about the appropriateness of exploring the needs addressed in the MAP guide	“How appropriate did you find the needs assessment conducted by the palliative care physician during the initial encounter?” Likert-type scale (1 = completely inappropriate; 5 = highly appropriate) assessed by phone	≥ 75% of patients/relatives give rating ≥ 3
**Participation**	Proportion of eligible patients who are assessed using the MAP guide and in whom more than 50% of the needs it considers are explored	Number of patients assessed using the MAP guide and in whom more than 50% of the needs it considers are explored *divided by* the total number of patients recruited	≥ 75% of eligible patients have more than 50% of needs assessed using the MAP guide
**Applicability**	Time to administer	Minutes	Less than 60 minutes required to assess patients’ needs using the MAP guide
**Utility**	Utility of the MAP guide as perceived by physicians	*“Could you please rate the perceived clinical utility of the MAP guide in evaluating your patients’ needs?”* Likert-type scale (1 = of no use at all; 5 = highly useful)	≥ 75% of physicians give rating ≥ 3
**Implementation**	Successful application of the MAP guide: Proportion of needs in each domain that are assessed	Researchers consult patients’ clinical records to ascertain what proportion of the needs considered by the MAP guide have been assessed	≥ 80% of the needs included in each domain of the MAP guide are recorded as having been assessed

### Data analysis

We first conducted a descriptive analysis of the full data set to determine whether each of the feasibility indicators had been fulfilled. This analysis was then repeated for the data sets corresponding to each of the three palliative care settings (outpatient, in-patient, domiciliary) to compare the degree of fulfillment across contexts.

### Qualitative procedure and data analysis

All physicians who used the MAP guide in a palliative care initial assessment were invited to participate in a focus group. The discussion covered five topics: [1] Difficulties encountered when using the MAP guide; [2] Changes in application between the first and final patient; [3] Adaptation: whether they considered including or excluding any needs to better suit their approach or setting; [4] Applicability: they were asked whether the MAP had been difficult to use with particular patients; and [5] Effectiveness: their views on its utility and future use.

The focus group was recorded and transcribed for content analysis [35] using ATLAS.ti 9. Initially, the transcription was read several times to gain an overview of its content. It was then coded, line by line, to identify units of meaning, which were then grouped into categories. Further analysis yielded three main themes. The final interpretation resulted from researcher consensus.

### Ethics committee approval

The study was approved by the Research Ethics Committee of the Universitat Internacional de Catalunya (ref. MED-2018-10), as well as by the review boards of the ten participant institutions. Participants gave written informed consent prior to the commencement of the study.

## Results

Twenty-four palliative care physicians applied the MAP guide with a total of 239 patients (mean age 70.8 years, range 43–96) across the different palliative care services, primarily in the outpatient setting (73%), followed by in-patients (15%) and domiciliary care (12%). The most prevalent forms of cancer were lung (17%) and colorectal (11%). Tables 3 and 4 show sociodemographic and clinical characteristics of study participants.

**Table 3:** Sociodemographic and clinical characteristics of patients who participated in the study (n = 239).

		Mean (SD)
**Age (years)**		70.8 (12.16)
		**n (%)**
**Gender**	Male	137 (57)
	Female	102 (43)
**Cancer**	Lung	40 (17)
	Colorectal	26 (11)
	Breast	15 (6)
	Prostate	18 (8)
	Urinary tract	13 (5)
	Female genital	15 (6)
	Head/neck	13 (5)
	Other	99 (41)
**Setting**	In-patient	35 (15)
	Outpatient	175 (73)
	Domiciliary	29 (12)

**Table 4:** Characteristics of palliative care physicians who participated in the study (n = 24).

		Mean (SD)
**Age (years)**		47.2 (9.43)
		**n (%)**
**Gender**	Male	13 (54)
	Female	11 (46)
**Setting**	In-patient	5 (21)
	Outpatient	17 (71)
	Domiciliary	2 (8)
**Region of Spain where employed**	Madrid	3 (13)
	Catalonia	19 (79)
	Navarre	2 (8)

### Quantitative component: Feasibility indicators

Table 5 shows the results obtained for each of the feasibility indicators.

**Table 5:** Results for the five feasibility indicators, both overall and in each of the three palliative care settings.

Indicator	Acceptability	Participation	Applicability		Utility	Implementation					
Definition of indicator	Opinion of patient and accompanying relative(s) about the appropriateness of exploring the needs addressed in the MAP guide (5-point Likert scale)	Proportion of eligible patients assessed using the MAP guide	Time to administer the MAP guide	Physicians’ perception of clinical utility (5-point Likert scale)		Successful application of the MAP guide: Proportion of needs in each domain that are assessed					
Fulfilled if…	≥ 75% of patients give rating ≥ 3	≥ 75% of eligible patients	< 60 minutes	≥ 75% of physicians give rating ≥ 3		≥ 80% of needs in each domain					
	Mean rating (1–5)	%	Minutes	Mean rating (1–5)		Clinical history	Physical symptoms	Functional & cognitive status	Psycho-emotional symptoms	Social issues	Spiritual/ existential concerns
Total sample (n = 239)	4.8	90	60	4.7		76%	85%	86%	72%	75%	74%
Outpatient (n = 175)	4.8	87	57	4.8		77%	85%	86%	69%	73%	66%
In-patient (n = 35)	4.8	100	45	4.7		73%	80%	90%	77%	81%	91%
Domiciliary (n = 29)	4.8	100	101	4.5		76%	87%	78%	83%	80%	96%

(a) **Acceptability**. A total of 239 patients and their accompanying relatives provided feedback on the acceptability. The overall mean rating was 4.8 (out of 5), with almost all patients (96%) and accompanying relatives (98%) giving a rating ≥ 3. These results are well above the established criterion (75% of patients/relatives give rating ≥ 3) and indicate a high degree of satisfaction with the MAP guide. The criterion was also easily surpassed when analyzing results for each of the three clinical settings: 96% of both patients and accompanying relatives in outpatients; 96% of both in-patients and patients under domiciliary care and 100% of accompanying relatives in both settings.
(b) **Participation.** The criterion for participation was fulfilled in 90% of cases overall, and in all patients assessed in the domiciliary and in-patient settings. Although, in the outpatient context, the criterion was not met in 23 of the 175 patients assessed (fewer than 50% of MAP needs were explored in these cases), the fulfillment rate (87%) is still well above the threshold established for the participation indicator.
(c) **Applicability.** The mean time required to administer the MAP guide was 60.45 (± 22.07) minutes, only slightly above the established threshold of less than 60 minutes. However, results differed across clinical settings. In both the outpatient and in-patient context the feasibility indicator for applicability was fulfilled (mean time of 57.02 and 45.74 minutes, respectively). By contrast, the mean time to administer the MAP guide to patients under domiciliary care was 101.11 minutes, and it was this result that increased the overall mean.
(d) **Utility.** The mean physician rating for perceived utility of the MAP guide was 4.7 (out of 5), with 95% of physicians giving a rating ≥ 3, well above the 75% required for fulfillment of the indicator. This feasibility indicator was also fulfilled in each of the three settings: outpatient, 94%; in-patient, 96%; domiciliary, 90%.
(e) **Implementation**. Overall, 78% of the needs included in the MAP guide were assessed, slightly below the established criterion of ≥ 80%. In terms of the six MAP domains, the threshold was surpassed in relation to the assessment of physical symptoms (85%) and functional and cognitive status (86%), but not in any of the other four domains, where between 72% and 76% of needs were assessed. Table 6 shows the overall percentage of needs that were explored in each domain, as well as the most and least commonly assessed need(s) in each case. Analysis of results by clinical setting showed that the implementation indicator was fulfilled in both the in-patient and domiciliary contexts (respectively, 82% and 83% of MAP needs were explored), compared with just 76% among outpatients.

**Table 6:** Implementation results for the six domains of the MAP guide, showing the most and least commonly assessed need(s) in each case.

MAP domains and needs		Total	Outpatient	In-patient	Domiciliary
**D1: Clinical history and medical conditions**		**76%**	**77%**	**73%**	**76%**
Most assessed	Diagnosis	100%	100%	100%	100%
	Current pharmacological treatment	97%	96%	100%	97%
Least assessed	Relevant medical history	45%	49%	17%	51%
	Complementary therapies	41%	42%	54%	17%
**D2: Physical symptoms**		**85%**	**85%**	**80%**	**87%**
Most assessed	Constipation	96%	96%	91%	100%
	Asthenia/fatigue	96%	95%	94%	100%
Least assessed	Pruritus	78%	79%	80%	69%
	Myoclonus	70%	71%	66%	69%
**D3: Functional and cognitive status**		**86%**	**86%**	**90%**	**78%**
Most assessed	Degree of functional dependence	97%	97%	100%	93%
	Cognitive status	93%	93%	91%	100%
Least assessed	Hallucinations	61%	63%	80%	28%
**D4: Psycho-emotional symptoms**		**72%**	**69%**	**77%**	**83%**
Most assessed	Mood/affect	94%	93%	97%	93%
	Current concerns	89%	88%	86%	93%
Least assessed	Anxiety	55%	53%	49%	72%
	Depression	53%	45%	80%	72%
**D5: Social issues**		**75%**	**73%**	**81%**	**80%**
Most assessed	Family tree/relationships	96%	95%	97%	97%
	Main caregiver	92%	89%	91%	100%
Least assessed	Perceived support by caregiver	61%	53%	60%	59%
	Need for social care	50%	47%	66%	52%
**D6: Spiritual and existential concerns**		**74%**	**66%**	**91%**	**97%**
Most assessed	Aspects that help the patient to cope	87%	84%	94%	97%
Least assessed	Patient’s values	66%	58%	83%	97%

### Qualitative component: Focus group

Of the 24 palliative care physicians who administered the MAP guide, 17 participated in the focus group, which lasted 90 minutes. Three themes emerged from the discussion of their experiences when applying the MAP guide: (a) More advantages than difficulties using the MAP guide; (b) Rapid learning despite an initial reluctance; and (c) Personal adaptations and suggestions for fine tuning the MAP guide. Illustrative quotations for each theme can be found in the S1 Table.

#### a) More advantages than difficulties using the MAP guide.

Most practitioners expressed that MAP was highly flexible and easy to integrate into actual clinical practice. The majority incorporated it into their daily assessment, adapting it to the time and staff resources available to the team. Moreover, they appreciated that using MAP allowed them to provide a much more comprehensive assessment than they normally did. The difficulties discussed by physicians related to exploring emotional and spiritual needs, implementing the guide within the time available to them in their work setting, and recording the needs explored in patients’ clinical records. Most of them felt they lacked training and confidence to explore a patient’s emotional and spiritual needs. Furthermore, in some palliative care teams, these issues were seen as the remit of the psychologist or social worker, and hence the physician did not usually include them as part of their initial assessment. As regards the question of time, many physicians had found it difficult to explore all the needs included in the MAP guide in less than 60 minutes. In the case of those working in domiciliary palliative care, 90 minutes were always set aside for the first appointment with patients, and hence there was little motivation to apply the MAP guide in less than 60 minutes. On the plus side, physicians considered that the MAP guide was flexible enough to allow them to apply it with all their patients.

#### b) Rapid learning despite an initial reluctance.

Physicians pointed out that they already had their own approach to assessment, which in many cases was similar to what was required of them by the MAP guide. As a result, they were initially reluctant to incorporate aspects of the MAP guide that they did not usually explore. Several of them recognized, however, that the MAP guide helped them to structure the initial assessment and to address the patient’s needs more comprehensively and efficiently. Furthermore, as they gained experience with using the MAP guide, they found it progressively easier to include aspects that they did not usually explore, adapting quickly to this new way of assessing palliative care needs.

#### c) Personal adaptations and suggestions for fine tuning the MAP guide.

All the physicians emphasized the utility of the MAP as a guide for exploring palliative care needs. However, their experience of using it also led them to identify certain changes that, in their view, would make it better adapted to clinical practice. Most of the proposed changes related to switching certain needs to a different domain, eliminating some of the less frequent symptoms, unifying some of the emotional needs, and focusing more on the patient’s information needs.

## Discussion

Our mixed-methods study guided by the RE-AIM framework [30] supports the feasibility of using the MAP guide to assess palliative care needs during the initial assessment in a standardized and comprehensive way. The MAP guide generally takes under 60 minutes to apply, and it is flexible enough to be used with patients across different settings. Indeed, while the guide sets out key areas that should be explored, physicians may go about this in whatever way they see fit. Importantly, several of the physicians considered that using the MAP guide had helped them to structure the initial assessment and to address the patient’s needs more efficiently, and the feedback from patients and families regarding its appropriateness was very positive. The guide also improved clinical records, enhancing communication within healthcare teams.

Regarding its application, physical symptoms were the most commonly explored domain. This is not surprising given that when developing the MAP guide, expert consensus was quickly reached about the importance of assessing this aspect [25]. Interestingly, however, it was also a domain that generated considerable debate in the focus group. Many of the physicians involved acknowledged that they already had their preferred method of assessing physical symptoms, which was usually based on the Edmonton Symptom Assessment System Scale (ESAS), one of the most widely used tools in this context [36], and they felt reluctant to change this. Unsurprisingly, therefore, physical symptoms such as pruritus or myoclonus, which feature in the MAP guide but not in the ESAS, were among the least commonly assessed. In fact, one of the suggestions made during the focus group was that applicability of the MAP guide could be improved by eliminating these less frequent physical symptoms [37–39].

Although the implementation results were generally positive, the assessment of needs in some domains was less comprehensive or consistent. For example, needs in the emotional, social, and spiritual domains were less commonly assessed and recorded than were those relating to physical symptoms or functional and cognitive status. Despite the fact that psychological, social and spiritual needs assessment are included in palliative care models [26,27], and are commonly reported in palliative care settings [40]. The challenge of exploring patients’ emotional or spiritual needs became apparent during the Delphi process conducted when developing the MAP guide [25], with many of the experts consulted acknowledging that they found this difficult, the perception being that they lacked the skills required to do so. However, evidence suggests that open communication with the patient about these topics strengthens the therapeutic relationship and benefits the patients [41,42]. Moreover, with proper training, professionals can effectively handle these conversations [41,43], providing essential support to patients throughout their process [44]. As Best et al. [45] point out, a failure to explore these needs may lead to a patient’s emotional or spiritual suffering going unaddressed, hence the importance of including this aspect in the initial palliative care assessment. One of the reasons given by some physicians in the focus group for their lack of experience or confidence in exploring patients’ emotional needs was that these issues were seen as the remit of the psychologist or social worker in their palliative care team. The problem is that not all palliative care teams have this multidisciplinary profile [46,47], and even in those which do, the psychologist or social worker is often not available on a full-time basis [48]; as a result, their attention has to be focused on those patients with the most urgent psychological needs, rather than offering an assessment in all cases. In our view, this highlights the need for palliative care physicians to ensure that they have the minimum level of communication and empathetic skills [8] required to conduct at least a preliminary exploration of a patient’s emotional and spiritual needs during the initial assessment. In the event that unmet psychological needs appear to be present, they may then request a more detailed assessment and, where necessary, intervention by an appropriate specialist.

Another fundamental question with regard to the feasibility of using the MAP guide in real clinical practice concerns time constraints. Conducting a meaningful clinical interview in which a patient’s needs can be comprehensively explored requires at least 60 minutes. Any circumstance which limits the time available will likely mean that physicians skip – either during the interview itself or subsequently when reporting in the patient’s clinical records – any needs that are perceived to be less common or important. In the present sample, those physicians working in domiciliary care were less affected by time constraints, as it was standard practice for 90 minutes to be set aside for the initial assessment. A study conducted to test the feasibility of a home-based palliative care intervention, suggested that efficiency could be optimized by incorporating novel technology (telehealth tools) [49]. This reflects a point made by some of the physicians in our focus group, who considered that the availability of a systematized (digital) template would make it easier to implement the MAP guide, reducing the time required to record information and making it less likely that they would overlook certain needs. During the focus group, it was suggested that one way of streamlining this process would be to ensure that a section detailing the needs addressed in the MAP guide was incorporated into the electronic health records of palliative care services. Good clinical record keeping is essential for tracking patients’ changing needs and for providing them with quality care through the efficient targeting of resources [50–52].

However, the MAP guide should not be considered or used simply as an electronic checklist for the assessment of patient needs, as this could hinder the therapeutic alliance [19].

The main limitation of this study is the sole inclusion of patients with advanced cancer, making it unclear how generalizable the findings are to other palliative care populations. Future research should explore the applicability of the MAP guide in the assessment of patients with, for example, amyotrophic lateral sclerosis, complex chronic diseases or hematological malignancies. Although the RE-AIM framework [30] was applied in this mixed-methods study, the maintenance dimension was not evaluated, as no longitudinal follow-up was conducted. Therefore, future studies should focus on assessing the long-term sustainability of the intervention within real-world palliative care practice. As for the study’s strengths, the inclusion of patients from across outpatient, in-patient, and domiciliary services, enabled us to confirm the applicability of the MAP guide in different contexts. Finally, consideration must be given to the suggested improvements to the guide that were made by physicians during the focus group.

## Conclusions

These results support the feasibility of using the MAP guide for a systematic and comprehensive assessment of palliative care needs across various settings. Initial reluctance among some physicians to explore all 47 needs was largely overcome once they gained experience of using it, and it was generally acknowledged that the MAP guide helped them to structure the initial assessment and to address the patient’s needs more comprehensively and efficiently. Patients and relatives also viewed the experience positively, considering the exploration of needs appropriate. By facilitating the assessment of multidimensional needs, the MAP guide can enhance equity of care by ensuring that professionals do not overlook care needs that, if unmet, might increase suffering and further undermine a patient’s quality of life. Future research should explore the clinical benefits and impact on quality of life outcomes associated with the use of MAP, as well as its potential integration into national guidelines.

## Supporting information

S1 Table Overview showing the main themes, sub-themes and illustrative quotations, that describe the experiences of palliative care physicians when applying the MAP guide. (DOCX)

## References

[1] World Health Organization. Palliative care. 2020. [Cited 3 June 2024]. https://www.who.int/news-room/fact-sheets/detail/palliative-care

[2] Hudson P, Collins A, Bostanci A, Willenberg L, Stepanov N, Philip J. Toward a systematic approach to assessment and care planning in palliative care: A practical review of clinical tools. Palliat Support Care. 2016;14(2):161–73. doi: 10.1017/S1478951515000565 26063219

[3] McMillan SC, Small BJ, Haley WE. Improving hospice outcomes through systematic assessment: a clinical trial. Cancer Nurs. 2011;34(2):89–97. doi: 10.1097/NCC.0b013e3181f70aee 20885302 PMC3036771

[4] Aslakson RA, Dy SM, Wilson RF, Waldfogel J, Zhang A, Isenberg SR, et al. Patient- and Caregiver-Reported Assessment Tools for Palliative Care: Summary of the 2017 Agency for Healthcare Research and Quality Technical Brief. J Pain Symptom Manage. 2017;54(6):961-972.e16. doi: 10.1016/j.jpainsymman.2017.04.022 28818633

[5] Goni-Fuste B, Crespo I, Monforte-Royo C, Porta-Sales J, Balaguer A, Pergolizzi D. What defines the comprehensive assessment of needs in palliative care? An integrative systematic review. Palliat Med. 2021;35(4):651–69. doi: 10.1177/0269216321996985 33648403

[6] Schepers SA, Sint Nicolaas SM, Haverman L. Real-world implementation of electronic patient-reported outcomes in outpatient pediatric cancer care. Psychooncology. 2017;26(7). 10.1002/pon.4242 27502744

[7] Jagsi R, Chiang A, Polite BN, Medeiros BC, McNiff K, Abernethy AP, et al. Qualitative analysis of practicing oncologists’ attitudes and experiences regarding collection of patient-reported outcomes. J Oncol Pract. 2013;9(6):e290-7. doi: 10.1200/JOP.2012.000823 23943890

[8] Suchman AL. A model of empathic communication in the medical interview. JAMA J Am Med Assoc. 1997;277:678. 9039890

[9] Wang T, Molassiotis A, Chung BPM, Tan JY. Unmet care needs of advanced cancer patients and their informal caregivers: a systematic review. BMC Palliat Care. 2018;17(1):1–29. 10.1186/s12904-018-0346-9 PMC6057056 30037346

[10] Tsatsou I, Konstantinidis T, Kalemikerakis I, Adamakidou T, Vlachou E, Govina O. Unmet Supportive Care Needs of Patients with Hematological Malignancies: A Systematic Review. Asia Pac J Oncol Nurs. 2020;8(1):5–17. doi: 10.4103/apjon.apjon_41_20 33426184 PMC7785074

[11] Hart NH, Crawford-Williams F, Crichton M, Yee J, Smith TJ, Koczwara B, et al. Unmet supportive care needs of people with advanced cancer and their caregivers: A systematic scoping review. Crit Rev Oncol Hematol. 2022;176:103728. doi: 10.1016/j.critrevonc.2022.103728 35662585

[12] Cochrane A, Woods S, Dunne S, Gallagher P. Unmet supportive care needs associated with quality of life for people with lung cancer: A systematic review of the evidence 2007-2020. Eur J Cancer Care (Engl). 2022;31(1):e13525. doi: 10.1111/ecc.13525 34729855

[13] Jang H, Lee K, Kim S, Kim S. Unmet needs in palliative care for patients with common non-cancer diseases: a cross-sectional study. BMC Palliat Care. 2022;21(1):151. doi: 10.1186/s12904-022-01040-0 36038840 PMC9426270

[14] Yadav S, Heller IW, Schaefer N, Salloum RG, Kittelson SM, Wilkie DJ, et al. The health care cost of palliative care for cancer patients: a systematic review. Support Care Cancer. 2020;28(10):4561–73. doi: 10.1007/s00520-020-05512-y 32440909

[15] Delgado-Guay MO, Parsons HA, Hui D, De la Cruz MG, Thorney S, Bruera E. Spirituality, religiosity, and spiritual pain among caregivers of patients with advanced cancer. Am J Hosp Palliat Care. 2013;30(5):455–61. doi: 10.1177/1049909112458030 22952129

[16] Balboni T, Balboni M, Paulk ME, Phelps A, Wright A, Peteet J, et al. Support of cancer patients’ spiritual needs and associations with medical care costs at the end of life. Cancer. 2011;117(23):5383–91. doi: 10.1002/cncr.26221 21563177 PMC3177963

[17] Kaasa S, Loge JH, Aapro M, et al. Integration of oncology and palliative care: A Lancet Oncology Commission. Lancet Oncology. 2018;19(11):e588–653. 10.1016/S1470-2045(18)30415-7 30344075

[18] Antunes B, Harding R, Higginson IJ, EUROIMPACT. Implementing patient-reported outcome measures in palliative care clinical practice: a systematic review of facilitators and barriers. Palliat Med. 2014;28(2):158–75. doi: 10.1177/0269216313491619 23801463

[19] Goni-Fuste B, Pergolizzi D, Monforte-Royo C, Julià-Torras J, Rodríguez-Prat A, Crespo I. What makes the palliative care initial encounter meaningful? A descriptive study with patients with cancer, family carers and palliative care professionals. Palliat Med. 2023;37(8):1252–65. doi: 10.1177/02692163231183998 37421148 PMC10604432

[20] Rubenstein LZ, Goodwin M, Hadley E, Patten SK, Rempusheski VF, Reuben D, et al. Working group recommendations: targeting criteria for geriatric evaluation and management research. J Am Geriatr Soc. 1991;39(9 Pt 2):37S-41S. doi: 10.1111/j.1532-5415.1991.tb05932.x 1885876

[21] Stuck AE, Siu AL, Wieland GD, Adams J, Rubenstein LZ. Comprehensive geriatric assessment: a meta-analysis of controlled trials. Lancet. 1993;342(8878):1032–6. doi: 10.1016/0140-6736(93)92884-v 8105269

[22] Rubenstein LZ, Alessi CA, Josephson KR. A randomized trial of a screening, case finding, and referral system for older veterans in primary care. J Am Geriatr Soc. 2007;55(2):166–74. 17302651 10.1111/j.1532-5415.2007.01044.x

[23] Ellis G, Gardner M, Tsiachristas A. Comprehensive geriatric assessment for older adults admitted to hospital. Cochrane Database Syst Rev. 2017;9(9):CD006211. 10.1002/14651858.CD006211.pub3 PMC6484374 28898390

[24] Sum G, Nicholas SO, Nai ZL, Ding YY, Tan WS. Health outcomes and implementation barriers and facilitators of comprehensive geriatric assessment in community settings: A systematic integrative review. BMC Geriatr. 2022;22(1). 10.1186/s12877-022-03024-4 PMC9052611 35488198

[25] Goni-Fuste B, Pergolizzi D, Monforte-Royo C, Alonso-Babarro A, Belar A, Crespo I, et al. Development of a Guide to Multidimensional Needs Assessment in the Palliative Care Initial Encounter (MAP). J Pain Symptom Manage. 2023;66(4):361-369.e6. doi: 10.1016/j.jpainsymman.2023.07.011 37468050

[26] Ferris FD, Balfour HM, Bowen K, Farley J, Hardwick M, Lamontagne C, et al. A model to guide patient and family care: based on nationally accepted principles and norms of practice. J Pain Symptom Manage. 2002;24(2):106–23. doi: 10.1016/s0885-3924(02)00468-2 12231127

[27] American Academy of Hospice and Palliative Medicine, Center to Advance Palliative Care, Hospice and Palliative Nurses Association, Last Acts Partnership, National Hospice and Palliative Care Organization. National Consensus Project for Quality Palliative Care: Clinical Practice Guidelines for Quality Palliative Care, Executive Summary. J Palliat Med. 2004;7(5):611–27. 15588352 10.1089/jpm.2004.7.611

[28] Craig P, Dieppe P, Macintyre S, Michie S, Nazareth I, Petticrew M. Developing and evaluating complex interventions: the new Medical Research Council guidance. Int J Nurs Stud. 2013;50(5):587–92. doi: 10.1016/j.ijnurstu.2012.09.010 23159157

[29] Pergolizzi D, Crespo I, Balaguer A, Monforte-Royo C, Alonso-Babarro A, Arantzamendi M, et al. Proactive and systematic multidimensional needs assessment in patients with advanced cancer approaching palliative care: a study protocol. BMJ Open. 2020;10(2):e034413. doi: 10.1136/bmjopen-2019-034413 32024792 PMC7045209

[30] Glasgow RE, Vogt TM, Boles SM. Evaluating the public health impact of health promotion interventions: the RE-AIM framework. Am J Public Health. 1999;89(9):1322–7. doi: 10.2105/ajph.89.9.1322 10474547 PMC1508772

[31] Creswell JW, Clark VLP. Designing and conducting mixed methods research. Sage Publication; 2017.

[32] Ferrell BR, Temel JS, Temin S. Integration of palliative care into standard oncology care: American Society of Clinical Oncology clinical practice guideline update. Journal of Clinical Oncology. 2017;35(1):96–112. 28034065 10.1200/JCO.2016.70.1474

[33] Bowen DJ, Kreuter M, Spring B, Cofta-Woerpel L, Linnan L, Weiner D, et al. How we design feasibility studies. Am J Prev Med. 2009;36(5):452–7. doi: 10.1016/j.amepre.2009.02.002 19362699 PMC2859314

[34] Jones F, Gage H, Drummond A, Bhalla A, Grant R, Lennon S, et al. Feasibility study of an integrated stroke self-management programme: a cluster-randomised controlled trial. BMJ Open. 2016;6(1):e008900. doi: 10.1136/bmjopen-2015-008900 26739723 PMC4716164

[35] Graneheim UH, Lundman B. Qualitative content analysis in nursing research: concepts, procedures and measures to achieve trustworthiness. Nurse Educ Today. 2004;24(2):105–12. doi: 10.1016/j.nedt.2003.10.001 14769454

[36] Hui D, Bruera E. The Edmonton Symptom Assessment System 25 years later: Past, present, and future developments. J Pain Symptom Manage. 2017;53(3):630–43. 28042071 10.1016/j.jpainsymman.2016.10.370 PMC5337174

[37] Boehlke C, Joos L, Coune B, Becker C, Meerpohl JJ, Buroh S, et al. Pharmacological interventions for pruritus in adult palliative care patients. Cochrane Database Syst Rev. 2023;4(2023):CD008320. doi: 10.1002/14651858.CD008320.pub4 37314034 PMC11339634

[38] Kelley AS, Morrison RS. Palliative Care for the Seriously Ill. N Engl J Med. 2015;373(8):747–55. doi: 10.1056/NEJMra1404684 26287850 PMC4671283

[39] Hui D, Dev R, Bruera E. The last days of life: symptom burden and impact on nutrition and hydration in cancer patients. Curr Opin Support Palliat Care. 2015;9(4):346–54. doi: 10.1097/SPC.0000000000000171 26509860 PMC4792116

[40] Lormans T, de Graaf E, van de Geer J, van der Baan F, Leget C, Teunissen S. Toward a socio-spiritual approach? A mixed-methods systematic review on the social and spiritual needs of patients in the palliative phase of their illness. Palliat Med. 2021;35(6):1071–98. doi: 10.1177/02692163211010384 33876676 PMC8189005

[41] Boström K, Dojan T, Rosendahl C, Gehrke L, Voltz R, Kremeike K. How do trained palliative care providers experience open desire to die-conversations? An explorative thematic analysis. Palliat Support Care. 2024;22(4):681–9. doi: 10.1017/S1478951522001006 35942616

[42] Rodin G, Zimmermann C, Rydall A, Jones J, Shepherd FA, Moore M, et al. The desire for hastened death in patients with metastatic cancer. J Pain Symptom Manage. 2007;33(6):661–75. doi: 10.1016/j.jpainsymman.2006.09.034 17531909

[43] Voltz R, Boström K, Dojan T, Rosendahl C, Gehrke L, Shah-Hosseini K, et al. Is trained communication about desire to die harmful for patients receiving palliative care? A cohort study. Palliat Med. 2022;36(3):489–97. doi: 10.1177/02692163211065671 34937431 PMC8972950

[44] Keall RM, Clayton JM, Butow PN. Therapeutic life review in palliative care: a systematic review of quantitative evaluations. J Pain Symptom Manage. 2015;49(4):747–61. doi: 10.1016/j.jpainsymman.2014.08.015 25261637

[45] Best M, Leget C, Goodhead A, Paal P. An EAPC white paper on multi-disciplinary education for spiritual care in palliative care. BMC Palliat Care. 2020;19(1). 10.1186/s12904-019-0508-4 PMC6964109 31941486

[46] Gamondi C, Larkin P, Payne S. Core competencies in palliative care: An EAPC white paper on palliative care education - Part 1. European Journal of Palliative Care. 2013;20(2):86–91.

[47] Radbruch L, Payne S. White paper on standards and norms for hospice and palliative care in Europe: Part 1. European Journal of Palliative Care. 2009;16(6).

[48] Spanish Palliative Care Society (SECPAL). Directorio de Recursos de Cuidados Paliativos en España 2015 [Directory of palliative care resources in Spain 2015]. 2016. https://www.secpal.org/wp-content/uploads/2022/01/monografia8_directorio.pdf

[49] Mayr FB, Plowman JL, Blakowski S. Feasibility of a home-based palliative care intervention for elderly multimorbid survivors of critical illness. American Journal of Critical Care. 2021;30(1):e12-31. 10.4037/ajcc2021117 PMC8010647 33385209

[50] Cammy R. Electronic health record tracking of psychosocial care in the context of serious illness: A narrative review. J Palliat Med. 2024. 10.1089/jpm.2023.0514 38484329

[51] Zupanc SN, Lakin JR, Volandes AE, Paasche-Orlow MK, Moseley ET, Gundersen DA, et al. Forms or Free-Text? Measuring Advance Care Planning Activity Using Electronic Health Records. J Pain Symptom Manage. 2023;66(5):e615–24. doi: 10.1016/j.jpainsymman.2023.07.016 37536523 PMC10592170

[52] Consolo L, Rusconi D, Colombo S. Implementation of the e-IPOS in home palliative cancer care: a quasiexperimental pilot study. Am J Hosp Palliat Care. 2024. doi: 10.1177/10499091241240667 PMC11636022 38504550

