# Supplementary material for: Implementation of a clinical interview guide to Multidimensional needs assessment in Palliative care (MAP): A multicenter mixed-methods feasibility study
Source: PLoS One. 2025 Jul 31;20(7):e0329354. doi: 10.1371/journal.pone.0329354 (PMC12312908; doi:10.1371/journal.pone.0329354)
Supplement: S1 Table — (DOCX) [file pone.0329354.s001.docx]

**S1 Table.** Overview showing the main themes, sub-themes and illustrative quotations, that describe the experiences of palliative care physicians when applying the MAP guide.

| Theme | Sub-theme | Quotations |
| --- | --- | --- |
| a) More advantages than difficulties using the MAP guide | Perception of improvement in visit structure | ‘I find the MAP structure very useful, [...] it allows you to have a sense of order when conducting the initial visits’. (P1) |
|  |  | ‘MAP is a tool that has enhanced my professional skills [...]. I believe my evaluation of the initial visit is now more comprehensive than it was prior to using MAP.’ (P2) |
|  |  | ‘MAP has greatly contributed to helping us organize our work and recognize the importance of conducting a thoroughly comprehensive first visit.’ (P3) |
|  |  | ‘We found it to be very useful. It helps guide the needs assessment and reminds us of the pending points to address in a second visit.’ (P4) |
|  |  | ‘MAP has helped me realize the importance of evaluating warning signs in all areas of the patient's health.’ (P5) |
|  |  | ‘MAP enables a highly comprehensive and well-rounded assessment.’ (P6) |
|  |  | ‘MAP functions as a guiding framework, reminding us of all the aspects to be assessed during an initial visit, while allowing flexibility to apply it fully or partially based on the patient's needs.’ (P7) |
|  |  | ‘A tool like MAP allows you to structure the first meeting with a patient in a very comprehensive way, and you adapt it depending on the patient’s needs, the time available, or the setting you are in. But this is the framework for the main or most important aspects we need to consider in a first interview.’ (P8) |
|  |  | ‘Furthermore, it standardizes the teams’ activities and provides a common language—especially for those who haven’t worked together for long. In other words, this is how we conduct the first visits, and when someone new joins, it ensures that, regardless of style, all initial patient evaluations are very similar.’ (P9) |
|  | Barriers to a truly comprehensive approach | “I don’t always explore the spiritual aspect, which may be due to a lack of training in spiritual care or not knowing how to properly approach and integrate it into the medical record during a patient’s first visit.’ (P10) |
|  |  | ‘Spirituality also needs to be addressed, but having the right tools and creating the proper environment are essential. It is important to continue promoting training and fostering the right atmosphere to enable this.’ (P9) |
|  |  | ‘We conduct a very superficial social assessment, and when some complexity is detected, social work steps in to perform a more thorough evaluation. Therefore, we do not explore this area in depth.’ (P5) |
|  |  | ‘Sometimes, a patient’s expectations during a first visit are very specific. Delving deeply into certain aspects right away can sometimes be overwhelming—even potentially causing some iatrogenic effects. It’s often better to focus on the specific issue the patient came for and spend more time on that, then complete the assessment in a second visit once there is more trust.’ (P11) |
|  |  | ‘The emotional and social aspects are well supported in our unit by psychology and social work. All patients are systematically seen by the psychologist and social worker, so they usually handle exploring those areas.’ (P6) |
|  |  | ‘I reflected on the fact that I had noted no low mood or anxiety, as I understood that these areas were not affected in the patient. During the visit, other areas needed to be explored more, so I didn’t ask further questions to delve deeply into the emotional domain.’ (P10) |
|  |  | ‘In the first visit, we try to conduct a multidimensional assessment. Obviously, if pain or poorly controlled symptoms are the main issue, the spiritual aspect is deferred to a second visit, as we understand the priority is to improve those symptoms.’ (P12) |
|  | Adjustment to the allotted visit time | ‘We have noticed some difficulty in adapting our usual first-visit approach to MAP, which has led us to increase the duration of the visit.’ (P15) |
|  |  | ‘We have 45 minutes for a first hospital visit, which is quite limited time to assess each of the areas that need to be considered in MAP.’ (P5) |
|  |  | ‘Our home care visits are already scheduled for 90 minutes, which has allowed us to incorporate MAP without any difficulty.’ (P1) |
|  |  | ‘Regarding time, I feel we’re always pressed, even though we’re scheduled for an hour, we often end up spending 90 minutes. Family members get involved, there’s a lot of distress, discussions with social work… there are always things that can improve the patient’s situation at that moment.’ (P10) |
|  |  | ‘Regarding time, everyone adapts MAP to the time they have. Ideally, it might take an hour or 75 minutes, but if you have 45 minutes, you complete the assessment in that time—always keeping in mind that the first visit is the most important and must be done well.’ (P2) |
| b) Rapid learning despite an initial reluctance | Similarities with routine clinical practice | ‘In home care, our visits and interventions involve social work, nursing, and medicine, which gives us more opportunity during the first visit to gather all the information. MAP essentially formalizes what we were already doing in the initial visit.’ (P1) |
|  |  | ‘We have a structured one-hour visit, where the assessment template includes about 70% or slightly more of the items agreed upon in MAP, which hasn’t posed any difficulty in clinical practice.’ (P9) |
|  |  | ‘(MAP) is very similar to what we usually do, which has made its integration into our practice easy.’ (P8) |
|  | Process of transformation and skill development | ‘The learning curve is quick. I remember the first MAP assessments I did—the first or second one, I still needed to refer to the notes, but by the fourth or fifth, I had already internalized the process…’ (P2) |
|  |  | ‘The learning has been significant—it makes you aware of the importance of good documentation and of structuring things in the best possible way. It also helps highlight certain items we know we should assess, but often don’t due to timing or other circumstances. MAP reminds us of the importance of taking a holistic view, and that having everything well-documented and doing things as thoroughly as possible is essential in our day-to-day work.’ (P12) |
|  |  | ‘The first visit in palliative care is very important, as it allows us to gather a great deal of information about the patient and connect with them across all dimensions—not just symptoms. [...] MAP helps us remember everything we need to assess, with the flexibility to understand that it doesn't all need to be covered in 45 minutes or an hour. Instead, it serves as a framework for the initial visits, depending on the patient and their needs.’ (P13) |
|  |  | ‘With continued use of MAP, one gains skill and expertise in assessing these needs in patients.’ (P14) |
|  |  | ‘MAP served as a prompt to start considering new aspects that I didn’t usually take into account during a first visit.’ |
| c) Personal adaptations and suggestions for fine tuning the MAP guide | Suggestions for improvement | ‘For instance, when exploring the spiritual dimension, we often find that the answers to different questions—what helps the patient cope, what gives meaning to life, or what values guide them—tend to revolve around the same theme: the family. This has led us to consider whether these items could be grouped together.’ (P17) |
|  |  | ‘Given that some symptoms are less commonly observed, it might be worth considering the removal of certain items.’ (P5) |
|  |  | ‘There are some symptoms we don’t usually ask about because they’re not very common in our patients. For example, if itching doesn’t seem relevant in a given case, I typically don’t bring it up.’ (P16) |
|  |  | ‘For example, in the history and pathology section, the item about the patient’s impact and expectations regarding the current illness—I see that more as part of the emotional domain.’ (P9) |
|  |  | ‘MAP could be adapted depending on the type of setting or the patient being evaluated. For example, with head and neck or lung cancer patients, our interview framework already includes some more specific questions tailored to those profiles, which aren’t part of the general interview. For instance, with lung patients, we ask more detailed questions about dyspnea or cough... MAP could be adjusted to suit different circumstances.’ (P8) |
|  |  | ‘I would include the assessment of the DAM in the first visit, since we know there are benefits to incorporating it at this stage.’ (P2) |
|  |  | ‘I believe it is similar to spirituality; I would include the DAM assessment in the first visit because it is relevant to patient care. Furthermore, I think healthcare professionals must embrace the responsibility to train themselves and acquire the skills to properly address it.’ (P15) |
